# Comprehensive analysis of bulk and single-cell RNA sequencing data reveals Schlafen-5 (SLFN5) as a novel prognosis and immunity

**DOI:** 10.7150/ijms.97975

**Published:** 2024-09-09

**Authors:** Yueh-Jung Wu, Chung-Chieh Chiao, Po-Kai Chuang, Chung-Bao Hsieh, Chou-Yuan Ko, Ching-Chung Ko, Chuan-Fa Chang, Tung-Yuan Chen, Ngoc Uyen Nhi Nguyen, Ching-Cheng Hsu, Tian-Huei Chu, Cheng-Chieh Fang, Hsuan-Yen Tsai, Hsien-Chun Tsai, Gangga Anuraga, Hoang Dang Khoa Ta, Do Thi Minh Xuan, Sachin Kumar, Sanskriti Dey, Fitria Sari Wulandari, Rosario Trijuliamos Manalu, Ngoc Phung Ly, Chih-Yang Wang, Yung-Kuo Lee

**Affiliations:** 1 Division of Colorectal Surgery, Department of Surgery, Kaohsiung Armed Forces General Hospital, Kaohsiung 80284, Taiwan.; 2 PhD Program for Cancer Molecular Biology and Drug Discovery, College of Medical Science and Technology, Taipei Medical University, Taipei 11031, Taiwan.; 3 Graduate Institute of Cancer Biology and Drug Discovery, College of Medical Science and Technology, Taipei Medical University, Taipei 11031, Taiwan.; 4 Institute of Biomedical Sciences, National Sun Yat-Sen University, Kaohsiung 80424, Taiwan.; 5 Division of General Surgery, Department of Surgery, Tri-Service General Hospital, Taipei 114202, Taiwan.; 6 Division of General Surgery, Department of Surgery, Kaohsiung Armed Forces General Hospital, Kaohsiung 80284, Taiwan.; 7 Division of Gastroenterology and Hepatology, Department of Internal Medicine, Kaohsiung Armed Forces General Hospital, Kaohsiung 80284, Taiwan.; 8 Department of Medical Imaging, Chi-Mei Medical Center, Tainan, Taiwan.; 9 Department of Health and Nutrition, Chia Nan University of Pharmacy and Science, Tainan, Taiwan.; 10 School of Medicine, College of Medicine, National Sun Yat-Sen University, Kaohsiung, Taiwan.; 11 Institute of Basic Medical Science, College of Medicine, National Cheng Kung University, Tainan 70101, Taiwan.; 12 Department of Medical Laboratory Science and Biotechnology, College of Medicine, National Cheng Kung University, Tainan 70101, Taiwan.; 13 Department of Internal Medicine, Division of Cardiology, The University of Texas Southwestern Medical Center, Dallas TX 75390, USA.; 14 Institute of Medical Science and Technology, National Sun Yat-Sen University, Kaohsiung 80424, Taiwan.; 15 Medical Laboratory, Medical Education and Research Center, Kaohsiung Armed Forces General Hospital, Kaohsiung 80284, Taiwan.; 16 Institute of Molecular Medicine, College of Medicine, National Taiwan University, Taiwan.; 17 Department of Life Sciences, National University of Kaohsiung, Taiwan.; 18 Department of Statistics, Faculty of Science and Technology, PGRI Adi Buana University, East Java, Surabaya 60234, Indonesia.; 19 Faculty of Pharmacy, Van Lang University, 69/68 Dang Thuy Tram Street, Ward 13, Binh Thanh District, Ho Chi Minh City 70000, Vietnam.; 20 Faculty of Biotechnology and Applied Sciences, Shoolini University of Biotechnology and Management Sciences, Himachal Pradesh, India.; 21 Department of Pharmacy, Faculty of Pharmacy, National Institute of Science and Technology, Jakarta, 12640, Indonesia.; 22 Natural Product Research Center, Korea Institute of Science and Technology (KIST), Gangneung 25451, Republic of Korea.; 23 Division of Bio-Medical Science and Technology, KIST School, University of Science and Technology (UST), Seoul 02792, Republic of Korea.

**Keywords:** colorectal adenocarcinoma (COAD), Schlafen (SLFN) family genes, cell metabolism, immune infiltration, tumor microenvironment, single cell technology

## Abstract

Recent advancements have elucidated the multifaceted roles of the Schlafen (SLFN) family, including SLFN5, SLFN11, SLFN12, SLFN13, and SLFN14, which are implicated in immunological responses. However, little is known about the roles of this gene family in relation to malignancy development. The current study aimed to explore the diagnostic and prognostic potential of Schlafen family genes in colorectal adenocarcinoma (COAD) through bioinformatics analysis. Leveraging advanced bioinformatics tools of bulk RNA-sequencing and single-cell sequencing, we conducted in-depth analyses of gene expressions, functional enrichment, and survival patterns of patients with colorectal cancer compared to normal tissue. Among Schlafen family genes, the transcription levels of SLFN5 in COAD tissues were significantly elevated and correlated with poor survival outcomes. Furthermore, SLFN5 regulated the immune response via Janus kinase (JAK)/signal transduction and activator of transcription (STAT)/interferon (IFN)-alpha/beta signaling. These chemokines in inflammation are associated with diabetes and metabolism, suggesting their involvement in altered cellular energetics for COAD progress. In addition, an immune cell deconvolution analysis indicated a correlation between SLFN5 expression and immune-related cell populations, such as regulatory T cells (Tregs). These findings highlighted the potential clinical significance of SLFN5 in COAD and provided insights into its involvement in the tumor microenvironment and immune regulation. Meanwhile, the drug discovery data of SFLN5 with potential targeted small molecules suggested its therapeutic potential for COAD. Collectively, the current research demonstrated that SFLN5 play crucial roles in tumor development and serve as a prospective biomarker for COAD.

## 1. Introduction

Colorectal adenocarcinoma (COAD) is the predominant subtype of colorectal cancer (CRC), which progresses slowly but ranks third among the leading causes of cancer-related deaths worldwide 1-3. CRC is a major public health concern, and a deeper understanding of treatment options is crucial for researchers investigating novel therapeutic strategies in cancer immunity. Chemotherapy remains a mainstay of CRC treatment, particularly for advanced or metastatic disease, such as 5-Fluorouracil (5-FU) 4, Capecitabine (Xeloda) 5, Irinotecan (Camptosar) and oxaliplatin (Eloxatin) 6. These drugs are often combined in various regimens depending on the specific stage and characteristics of the CRC. For patients with microsatellite instability (MSI) tumors 7-9, a subset of CRC characterized by high rates of mutations, immunotherapy has emerged as a promising treatment option 10. MSI tumors are more likely to be recognized by the immune system due to the presence of these mutations, making them more susceptible to immune checkpoint inhibitors, such as pembrolizumab (Keytruda) and nivolumab (Opdivo) work by blocking molecules that normally dampen the immune response, this allows T cells to recognize and attack cancer cells more effectively 11.

The Schlafen (SLFN) gene family, named after the German word schlafen meaning sleeping, was initially identified as a group of growth-regulatory genes in mice that exhibited differential regulation during thymocyte development and T cell activation 12-14. In humans, there are several SLFN family members, including SLFN1, SLFN5, SLFN11, SLFN12, SLFN12L, SLFN13, and SLFN14, were originally identified for their roles in mediating antiviral responses; however, they were also recently reported to be involved in various cellular processes including cell cycle regulation, cell proliferation, DNA repair, and protein folding 15-17. Such diverse functions of SLFNs suggest their potential significance in malignant diseases. However, the comprehensive roles of SLFN family genes in pan-cancer analysis are yet to be fully clarified.

Advancements in bioinformatics have revolutionized our ability to dissect the molecular intricacies of cancers 18-20. Integrating large-scale omics data, including genomics, transcriptomics, and proteomics, has provided unprecedented insights into the complex landscape of genetic alterations and signaling pathways associated with different tumor stages 21-23. The ability to identify dysregulated pathways, gene expression clusters, and potential therapeutic targets has been facilitated by bioinformatic tools, thus contributing to the development of molecular subtyping strategies and assisting the provision of personalized treatment approaches 24-29. Bioinformatic analyses have revealed that among SLFN family members, SLFN5 stands out as being uniquely overexpressed in COAD compared to normal tissues. Therefore, this study prioritized SLFN5 for further examination, given its significant expression differences and potential biological importance in COAD.

## 2. Materials and Methods

### 2.1 Tissue microarray and immunohistochemical analyses

To construct a Tissue microarray (TMA) and immunohistochemical (IHC) analyses, 1.5-mm-diameter cores were extracted from 52 COAD patient-derived paraffin-embedded specimens, curated in the pathology archives of the Kaohsiung Armed Forces General Hospital, with approval by the Institutional Review Board (KAFGH-IRB: 111-041). Representative regions encompassing normal, neoplastic and adjacent non-neoplastic epithelial tissues were selected based on histological evaluation of hematoxylin and eosin (H&E)-stained sections. Sections of 4-μm thickness were prepared from compiled TMA blocks, followed by sequential deparaffinization in xylene and rehydration through graded ethanol solutions. The rehydrated sections underwent antigen retrieval in 10 mM sodium citrate buffer. Endogenous peroxidase activity was mitigated by treating sections with a 3% hydrogen peroxide solution in methanol for 30 min. After peroxidase blocking, tissue sections were incubated with a primary antibody that specifically targeted SLFN5 (clone no. HPA017760, Merck, Darmstadt, Germany) while being maintained at 4 °C overnight. This step was followed by incubation with an appropriate secondary antibody, following the manufacturer's specifications. Antigen-antibody complexes were detected using the 3,3'-diaminobenzidine tetrahydrochloride (DAB) chromogen, while employing a Vectastain ABC kit (Vector Laboratories, Burlingame, CA, USA) for 1 min. The specificity of the anti-SLFN5 antibody was validated by implementing both negative and positive control experiments by Hao-Long Biotechnology-Ltd., Kaohsing City, Taiwan, as we previously described 30-32.

### 2.2. Bioinformatic and functional enrichment analyses

In this study, gene expression data of colorectal tissues were obtained from The Cancer Genome Atlas (TCGA) patients, paired with transcriptomic data of normal colon tissues obtained from the GTEx platform 33, to conduct a differential gene expression (DEG) analysis of the SLFN gene family among various cancer types 34. To gain insights into the functional implications of SLFN5-regulated genes, we utilized the clusterProfiler R package 35, Omics Playground v.3.4.1 36, and SRplot platform 37, which were powerful tools for conducting functional enrichment analyses. Briefly, The Cancer Genome Atlas Colon Adenocarcinoma (TCGA-COAD) patients were divided into two groups based on the median expression of the SLFN5 gene. Lists of significant DEGs from these cohorts were then input into an algorithm to investigate biological processes, molecular functions, and cellular components 38. To provide a more comprehensive analysis, we ranked the DEG gene list and conducted a gene set enrichment analysis (GSEA) using the Hallmark database 39. For pathway analysis, we initially downloaded genes co-expressed with SLFN5 in COAD patients from TCGA via the cbioportal platform. Subsequently, MetaCore was employed to construct networked pathways derived from an input gene list to explore biological processes 40-42.

### 2.3 Survival analysis

To investigate the association between SLFN5 expression and the overall survival of COAD patients from TCGA, we utilized the Kaplan-Meier (KM) plotter within the UALCAN platform 43. UALCAN allows us to analyze pre-computed KM survival curves for genes of interest across various cancer types, including COAD. In the current analysis, we focused on COAD patients and compared the survival of patients with high and low SLFN5 expression levels based on the UALCAN predefined cut-off. The KM plot generated by UALCAN provides hazard ratios (HRs) with 95% confidence intervals (CIs) and a log-rank p-value to assess the statistical significance of the observed difference in survival between the two groups. This approach leverages the robust and well-annotated TCGA data, eliminating the need for separate data retrieval and analysis from original datasets 44-46.

### 2.4 DNA methylation and Cancer Cell Line Encyclopedia analyses

In this study, we performed DNA methylation analysis to examine variations in methylation patterns of SLFN5 in COAD patients using data from the TCGA dataset. To achieve this, we utilized several methylation databases by MethSurv 47, which is a comprehensive tool for investigating the expression and prognostic patterns of single CpG methylation sites of SLFN5 in COAD 48-50. In conjunction with investigating SLFN5 mRNA expression levels from the TMNplot database 51, we further examined its expression levels in different cell lines using the Cancer Cell Line Encyclopedia (CCLE) database. CCLE provides comprehensive access to pharmacologic and genetic characterizations of diverse human cancer models, while the RNA-Seq aligned reads tool was utilized on 60 independent COAD cell lines using default settings, as previously described 52-54.

### 2.5. Gene set signature analysis and immune deconvolution

The COAD patients from TCGA were grouped based on the median expression level of SLFN5 into high and low groups. The GSEA was used to identify groups of genes that were more or less active depending on SLFN5 expression. Results were considered to be statistically significant if they met specific criteria: a false discovery rate (FDR) of 0.25, a normalized enrichment score (NES) of > 1.5 in absolute value, and a nominal p-value of < 0.05. A positive NES indicated that genes at the top of the list were more active in patients with higher SLFN5 expression 55-59. These comprehensive analyses provide valuable information for identifying potential biomarkers, therapeutic targets, and personalized treatment strategies in cancer research.

### 2.6. Single-cell RNA-sequencing and antibody-based protein profiling using the Human Protein Atlas

The Human Protein Atlas (HPA) encompasses a comprehensive tissue-based map of the human proteome 60, and single-cell type transcriptomics for different types of cancers 61. Therefore, we leveraged the HPA to methodically evaluate the mRNA and protein levels pertaining to SLFN5 expression across both normal and malignant COAD tissues. Meanwhile, to investigate how SLFN family members contribute to the heterogeneity of COAD, we conducted single-cell analysis of colon tissues. Results are presented through a dendrogram illustrating relationships between different cell types, a gene cluster Uniform Manifold Approximation and Projection (UMAP) plot depicting gene relationships across cell types, and a summary of genes elevated in each cell type 62-66.

### 2.7. Drug sensitivity analysis

To evaluate the sensitivity of SLFN5 to various drugs, we utilized the Genome Set for Cancer Analysis (GSCA), this platform including Genomics of Drug Sensitivity in Cancer (GDSC) and the Cancer Therapeutics Response Portal (CTRP). Using GSCA, we identified several small-molecule compounds exhibiting significant sensitivity correlations with SLFN5, providing a robust framework for exploring new therapeutic avenues 67-70.

### 2.8. Statistical analysis

In this study, all statistical analyses were performed using R version 4.1.3. The significance of differences among various phenotypic groups was evaluated with the Wilcoxon test, which is a non-parametric statistical test suitable for comparing two independent samples. To address multiple testing, p values from the Wilcoxon test were adjusted using the FDR method. A p-value of < 0.05 was considered statistically significant, as we previously described 71-73.

## 3. Results

### 3.1. Differential expression levels of SLFN5 in COAD

**Figure 1:**
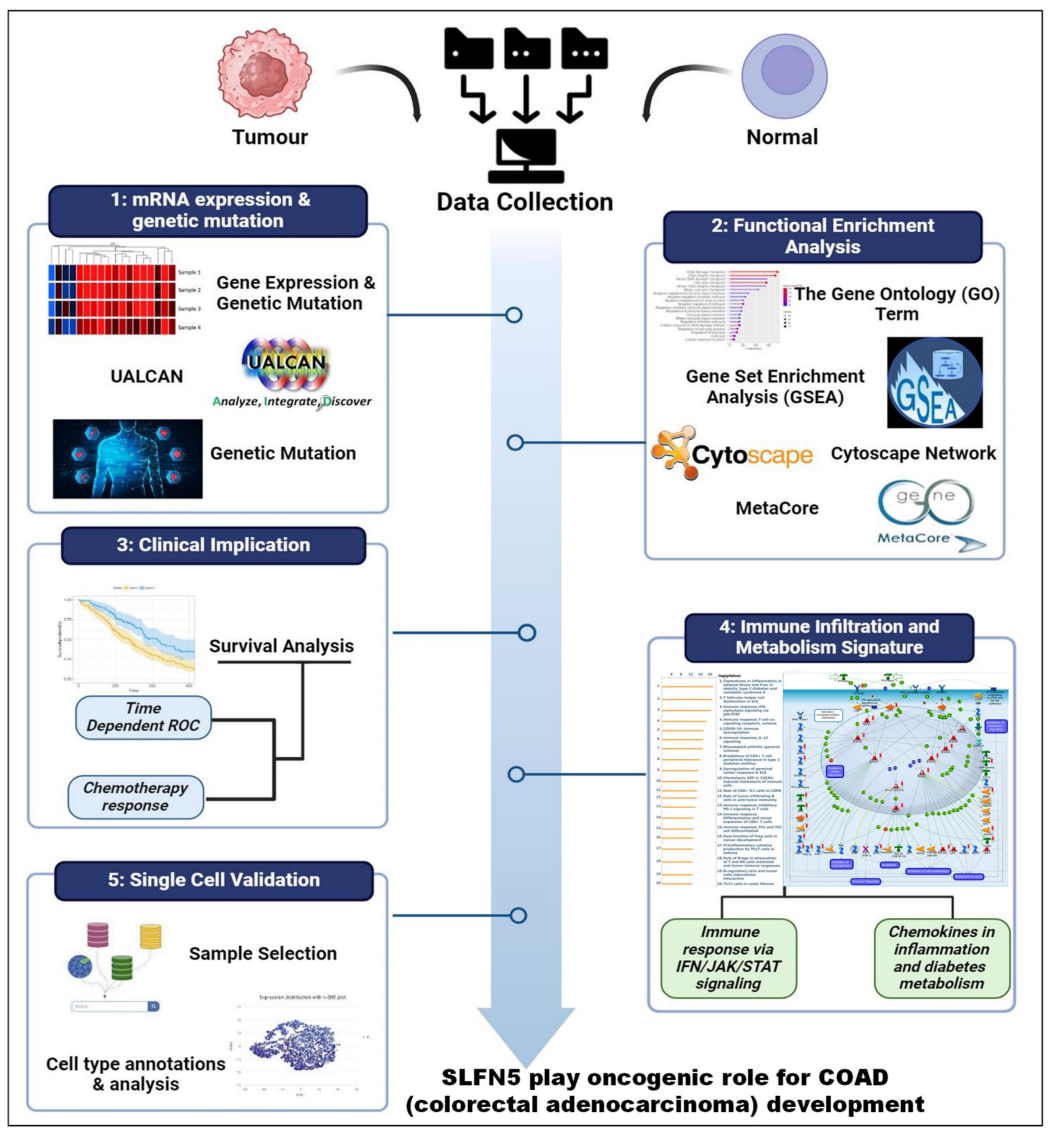
Overview of the workflow of the study.

**Figure 2:**
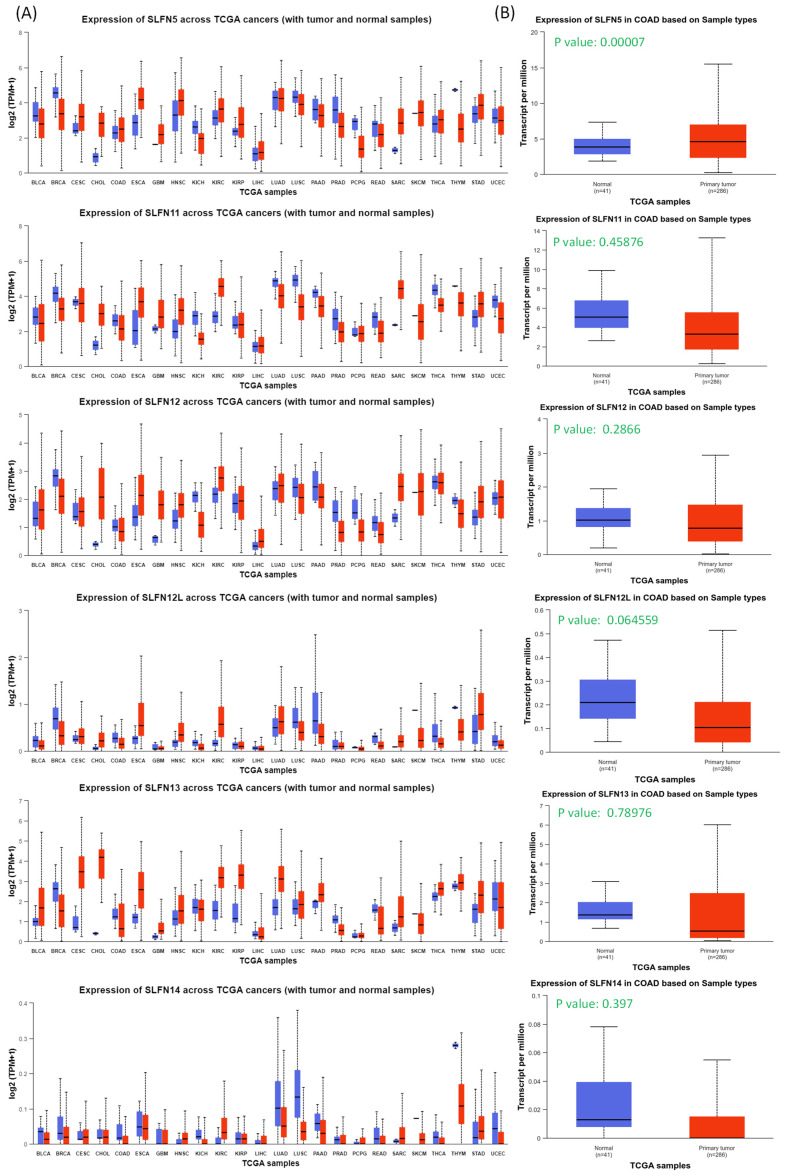
Pan-cancer analysis of Schlafen (SLFN) family genes from TCGA patients. (A) Transcript levels of SLFN family genes in normal and cancer tissues from TCGA patients via the UALCAN platform. (B) Box plots indicate SLFN family mRNA expression in TCGA dataset, and only SLFN5 had high expression levels in COAD patients (p < 0.05 was considered significant).

**Figure 3:**
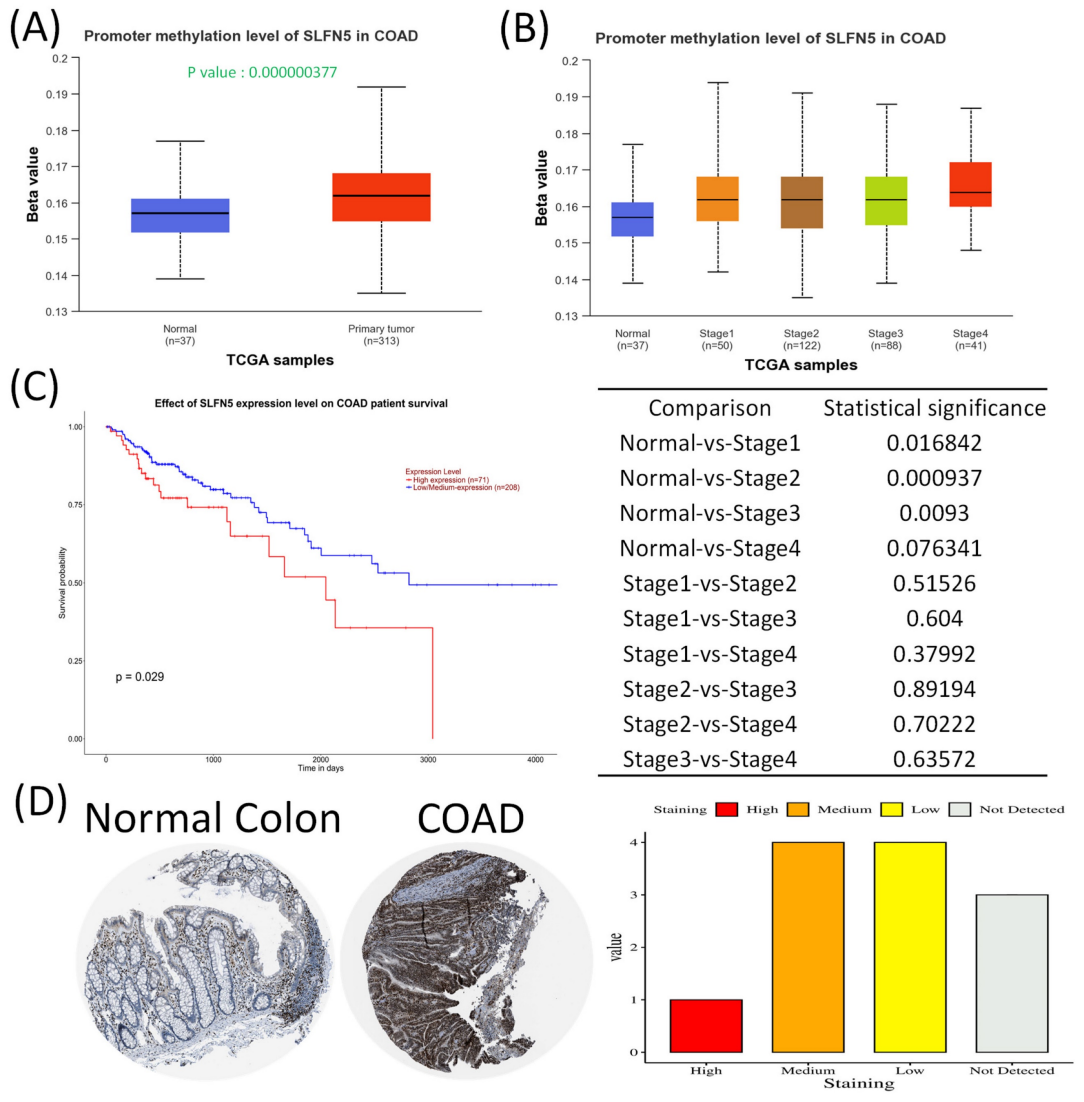
Schlafen 5 (*SLFN5*) plays an oncogenic role in COAD development. (A-B) SLFN5 promoter methylation levels in COAD were determined by a patient's tumor stage (p < 0.05). (C) Kaplan-Meier plot of SLFN5 expression in COAD patients for an overall survival analysis from TCGA patients via the UALCAN platform. (D) Analysis of SLFN5 in COAD specimens using the Human Protein Atlas (HPA). Protein levels of SLFN5 were examined in clinical COAD specimens from the HPA. IHC images of SLFN5 illustrate staining intensities. The HPA provided IHC images and patient information, including both normal and tumor samples. Bar charts present quantified IHC staining in COAD specimens, allowing for a comparative analysis.

In this study, leveraging advanced bioinformatic tools using bulk sequencing and single-cell sequencing, we conducted in-depth analyses of gene expressions, functional enrichment, and survival patterns (Figure 1). First, expressions of SLFN family genes in a pan-cancer analysis were analyzed by comparing TCGA cancer patients to normal tissue. Expressions of SLFN family genes varied across different types of cancer and relevant normal samples (Figure 2A). Furthermore, we assessed transcriptional levels of SLFN family genes between COAD patients and normal tissue. The data revealed that only transcription levels of SLFN5 in COAD tissues were significantly higher than normal tissue (Figure 2B). Subsequently, further analysis suggested that methylation levels of the SLFN5 promoter were significantly positively associated with malignant stages of COAD patients (Figure 3A, B). Next, KM curves and log-rank test outcomes also indicated that elevated expression of SLFN5 was linked to a shorter overall survival (OS) rate in COAD patients (Figure 3C).

**Figure 4:**
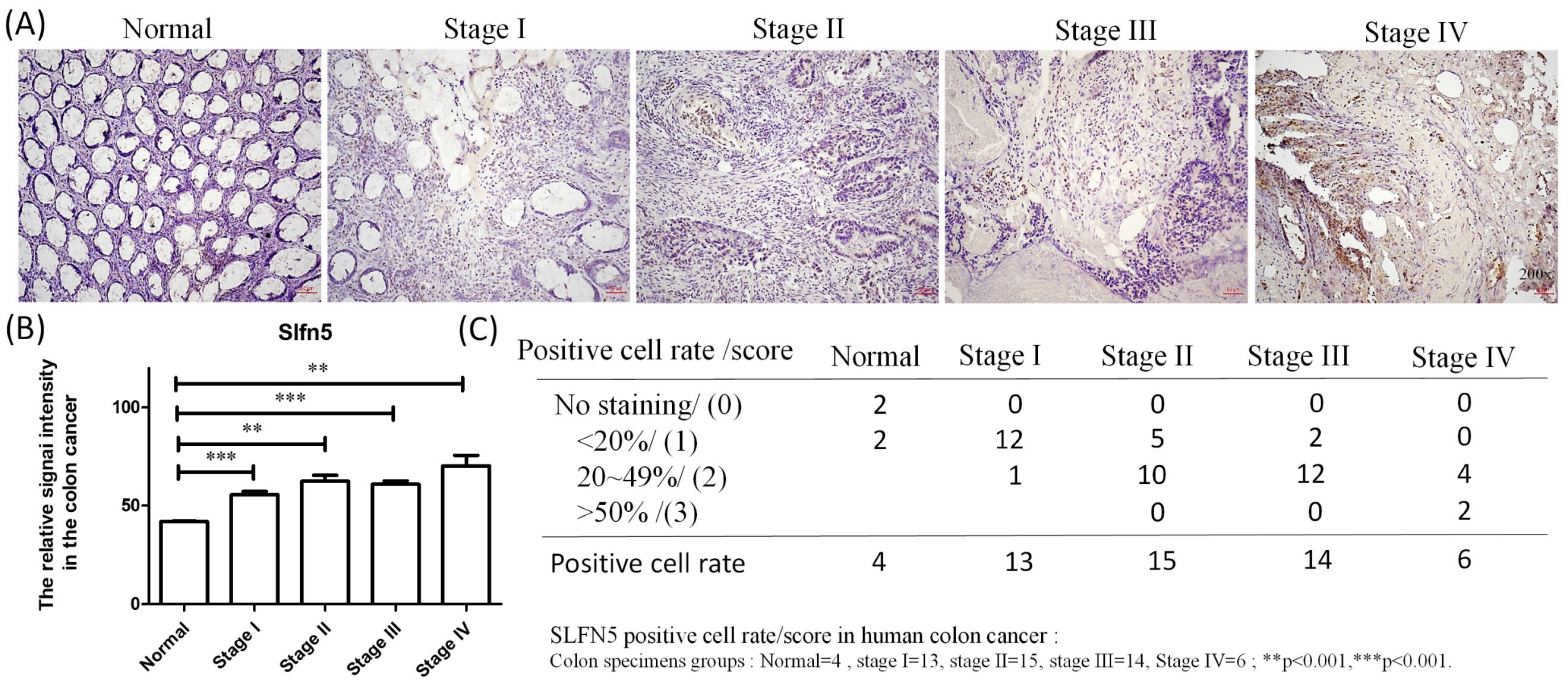
IHC expression of Schlafen 5 (SLFN5) in patients at different stages of colorectal adenocarcinoma (COAD) at Kaohsiung Armed Forces General Hospital. (A) Immunohistochemical staining showing the expression of SLFN5 in normal colon tissues, and COAD tissues at different stages (I-IV). (B-C) Quantitative analysis of SLFN5 positive cell rates in normal and various stages of COAD. The bar graph represents the percentage of positive cells in each group: Normal (n=4), Stage I (n=13), Stage II (n=15), Stage III (n=14), and Stage IV (n=6). Statistical significance was noted with **p<0.001 and ***p<0.001.

IHC analyses are a significant method in cancer detection as they allow visualization of antigen-antibody responses within tumors, which is particularly useful for identifying upregulated antigens. The HPA database provides IHC images of SLFN5 in COAD tissues, illustrating expression patterns in both normal colon and COAD tissues. IHC-stained images depict the intensities of antibodies in both COAD and adjacent normal tissues, with a bar chart under each IHC-stained image indicating the IHC staining intensity for SLFN5 (high, medium, low, and not detected) (Figure 3D). On the other hand, clinical validation results of IHC assays for SLFN5 protein expression by COAD patients at Kaohsiung Armed Forces General Hospital (Figure 4). Both IHC analytical results confirmed increased protein expression of SLFN5, consistent with the above mRNA differential expression analyses.

### 3.2. Bulk and single-cell RNA-Seq analysis using the HPA

**Figure 5:**
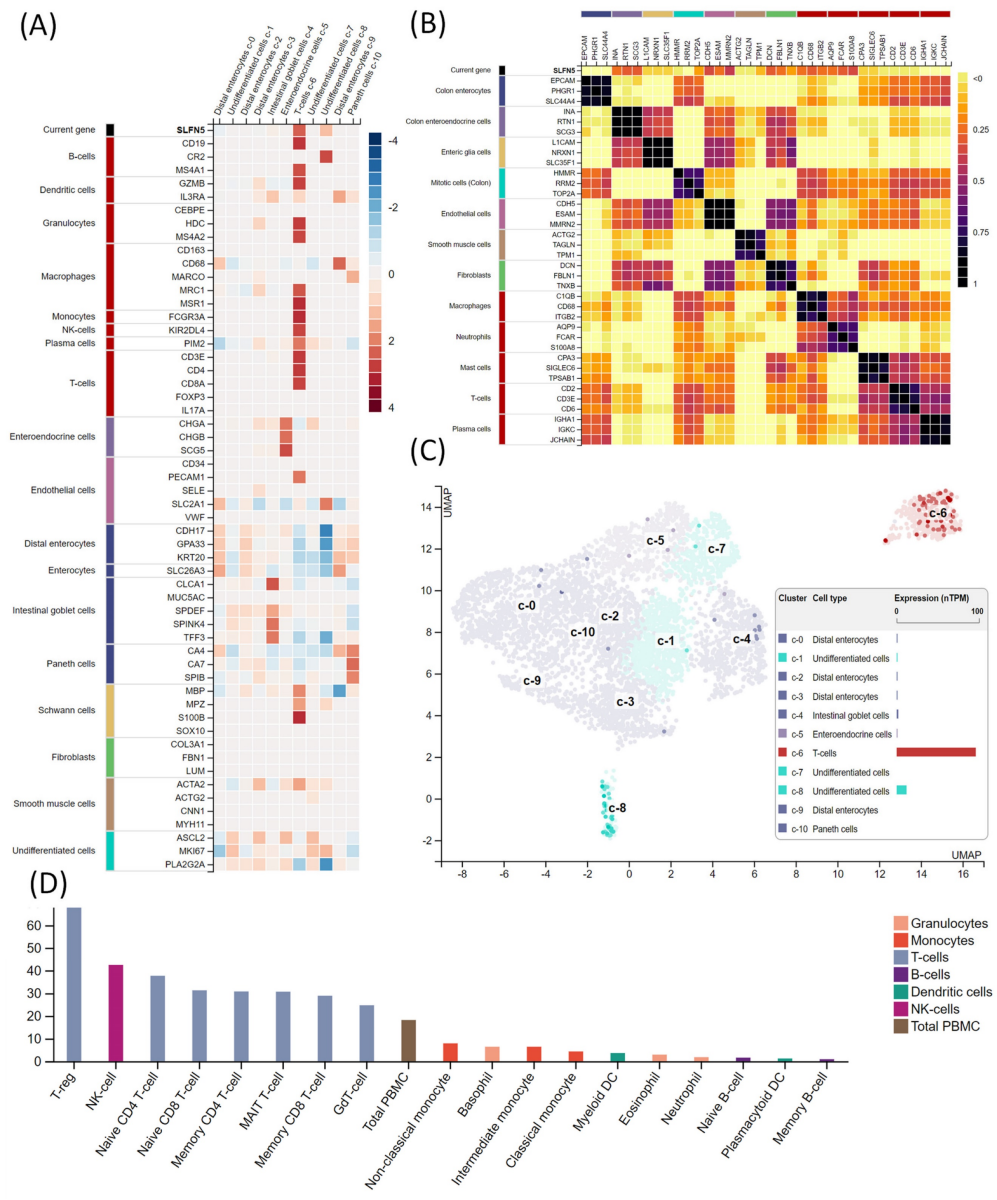
Analysis of Schlafen 5 (SLFN5) in tumor microenvironment characteristics in colorectal adenocarcinoma (COAD). (A) Heatmap shows SLFN5 expression (on top) and well-known cell-type markers in different single cell-type clusters of this tissue. The panel on the left shows which cell type each marker is associated with. Color coding is based on cell type groups, each consisting of cell types with common functional features. (B) Correlations of SLFN5 in a pan-cancer analysis. (C) RNA expressions in the single-cell type clusters identified in this tissue were visualized by a UMAP plot (left) and a bar chart (right). (D) Immune cell expression of SLFN5 as obtained from the Human Protein Atlas.

Given the heterogeneity of COAD, we performed single-cell analysis to characterize the transcriptomic expression of SLFN5 within different cell clusters. A heatmap (Figure 5A) quantitatively displays SLFN5 expression levels in conjunction with established cellular markers across distinct single-cell clusters identified within COAD tissues. Accompanying the heatmap is an annotated legend categorizing each marker according to its corresponding cell type, employing a systematic color-coding scheme to delineate groups of cell types possessing analogous functional attributes. Herein, we comprehensively examined SLFN5 expression patterns across diverse cancer types to ascertain commonalities and distinctions in its expression profile. This assessment aimed to unveil the broader oncological significance of SLFN5, extending beyond its role in COAD (Figure 5B). Expression dynamics of SLFN5 at the single-cell level are depicted through a dual-modality approach, incorporating a UMAP plot for a distribution analysis (left) and a quantitative bar chart for an expression level assessment (right). These methodologies collectively elucidated intricate expression patterns of SLFN5 within the heterogeneous cellular landscape of the COAD tumor microenvironment (TME) (Figure 5C).

In particular, an immune cell deconvolution analysis indicated a correlation between SLFN5 expression and immune-related cell populations, such as regulatory T cells (Tregs). A critical process in tumor development is forming an immunosuppressive state within the TME. Tregs are an essential type of suppressive immune cell. Research has demonstrated that Tregs can inhibit the function of effector T cells through various mechanisms, including cell surface inhibitors, inhibitory cytokines, and direct cytotoxicity. A growing body of preclinical data suggests that depleting Tregs or inhibiting their function can promote tumor regression. Additionally, apoptotic Tregs may exacerbate immunosuppression, leading to ineffective immunotherapies targeting immunosuppressive pathways. Meanwhile, the inclusion of single-cell expression data aimed to shed light on potential interactive dynamics between SLFN5 expression and immunological constituents within the COAD tumor milieu, thereby enriching our understanding of the immunobiological underpinnings associated with SLFN5's presence (Figure 5D).

### 3.3. Methylation and CCLE analysis

**Figure 6:**
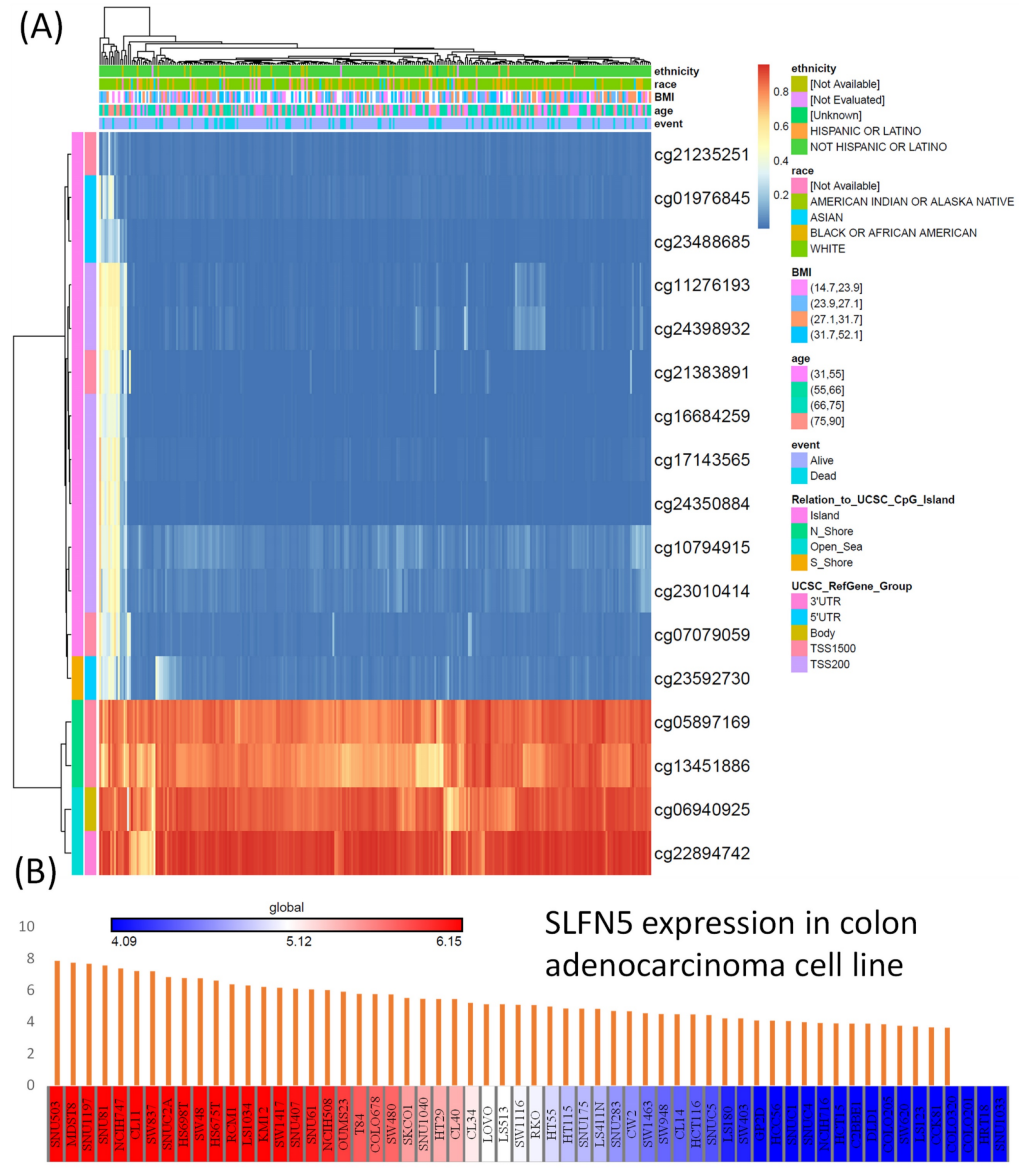
DNA methylation of Schlafen 5 (*SLFN5*), and transcript expression levels of *SLFN5* in different colorectal cancer cell lines (A) Heatmap of DNA methylation expression levels of *SLFN5* in TCGA colorectal adenocarcinoma (COAD) patients. (B) RNA-sequencing analysis of *SLFN5* mRNA in different colorectal cancer cell lines from the Cancer Cell Line Encyclopedia (CCLE). For each cancer cell line, high *SFLN5* expression is indicated in red (left), while low expression is indicated in blue (right).

For the DNA methylation analysis, a heatmap was created to show locations of DNA methylation in SLFN5 in COAD. Methylated CpG sites were identified for SLFN5, among which one site had high expression levels. Out of all of the sites, cg05897169, cg13451886, cg06940925, and cg22894742 showed higher levels of DNA methylation (Figure 6A). On the other hand, the RNA-Seq analysis from the CCLE was employed to evaluate SLFN5 mRNA expression levels across different CRC cell lines. The color gradient within the presentation, ranging from red (indicating high expression) to blue (indicating low expression), provides a comparative view of SLFN5 transcript abundances in each cancer cell line, thus offering insights into its regulatory landscape across diverse cellular backgrounds (Figure 6B).

### 3.4. GSEA and gene ontology (GO) analysis

**Figure 7:**
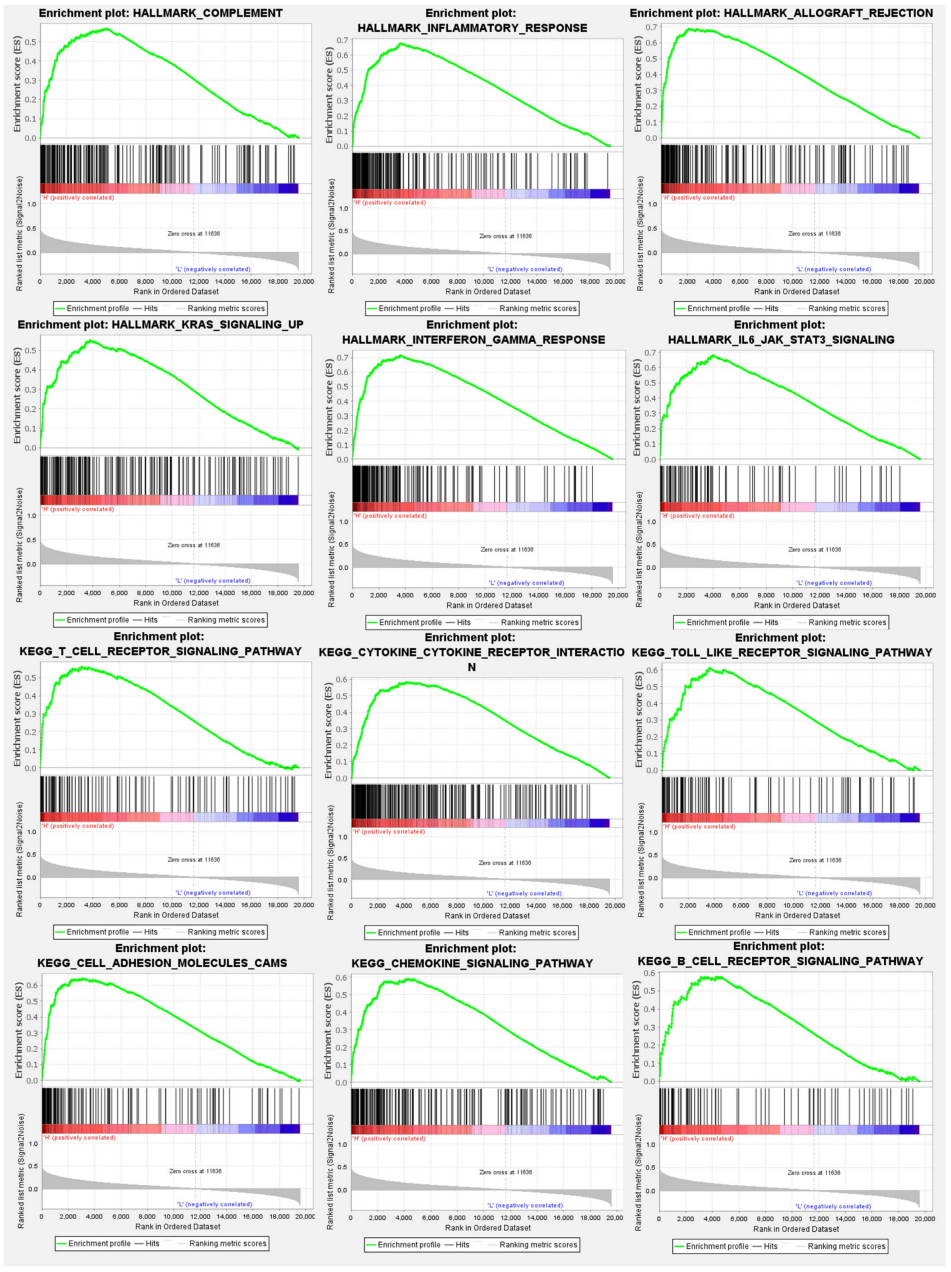
Hallmark signaling pathway analysis of Schlafen 5 (SLFN5) in colorectal adenocarcinoma (COAD). TCGA colorectal cancer patients were stratified into two groups based on the median SLFN5 expression value. A gene set enrichment analysis (GSEA) was then performed on these two groups using the Hallmark database. Results of the analysis revealed relevant enriched pathways in COAD groups with elevated SLFN5 expression.

Expression levels of the SLFN5 gene were divided into high- and low-expression cohorts based on median expression values from TCGA-COAD patients. A subsequent differential expression analysis utilized the DESeq2 computational method within the R/Bioconductor ecosystem. This analysis facilitated the identification of DEGs correlated with SLFN5 expression levels. The derived gene expression disparities provided the foundation for a subsequent GSEA, employing Hallmark gene sets within the fgsea package of R/Bioconductor. GSEA results illuminated statistically significant gene sets, characterized by their p values and NESs, thereby elucidating functional implications and biological relevance of these gene sets with SLFN5 expression disparities in COAD (Figure 7).

**Figure 8:**
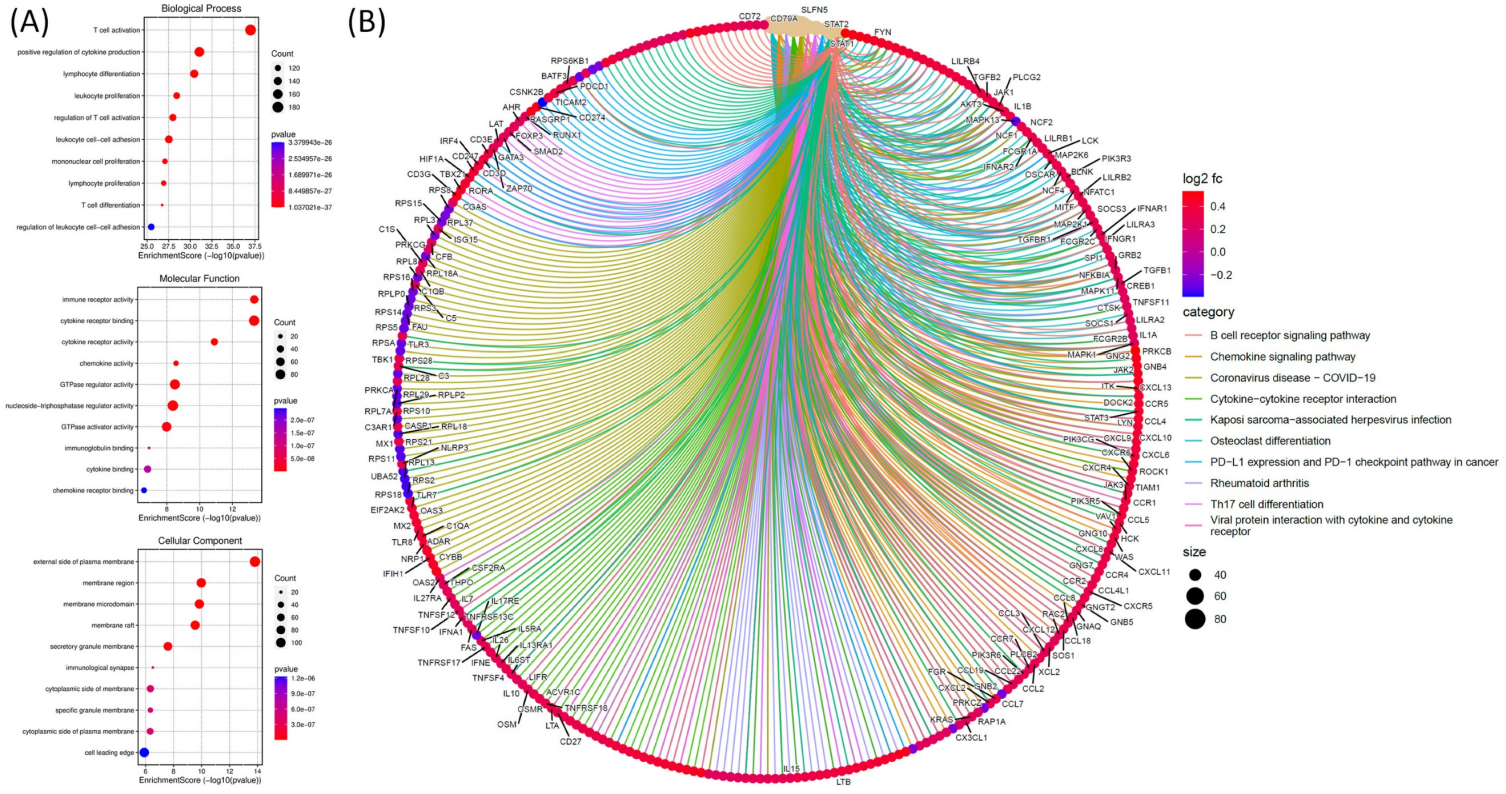
Functional and pathway enrichment analyses were performed specifically on genes co-expressed with Schlafen 5 (*SLFN5*) in colorectal adenocarcinoma (COAD) TCGA patients. (A) The analysis involved gene ontology (GO) terms, including biological processes, cellular components, and molecular functions. Circle sizes in the visual representation of the results indicate the number of genes associated with each function, while colors of the bubbles correspond to *p* values, providing information about the statistical significance of the enrichment. (B) Pathway analysis of *SLFN5* co-expressed genes in the KEGG database.

For the GO analysis, functional and pathway enrichment analyses were performed on genes co-expressed with SLFN5 in COAD using TCGA data. The GO analysis was conducted to explore biological processes, cellular components, and molecular functions associated with genes co-expressed with SLFN5 in COAD. The graphical representation utilizes various circle sizes to represent the number of genes involved in each specific GO term and quantify their biological impacts. Additionally, color gradations within these graphical elements signify p values associated with GO terms, providing a statistical measure of the significance of enrichment. This analytical approach aids in decoding complex biological narratives underpinning the network of genes co-expressed with SLFN5 (Figure 8A). For the Kyoto Encyclopedia of Genes and Genomes (KEGG) pathway enrichment analysis, an examination was undertaken to identify and analyze pathways enriched within the cohort of SLFN5 co-expressed genes, utilizing comprehensive datasets available within the KEGG database. This pathway enrichment analysis shed light on the broader biological pathways and mechanisms potentially modulated by the SLFN5 co-expression network, offering insights into systemic biological interactions and pathways implicated in COAD's etiology and progression (Figure 8B).

### 3.5. Exploring biological pathways and disease associations through the MetaCore database

**Figure 9:**
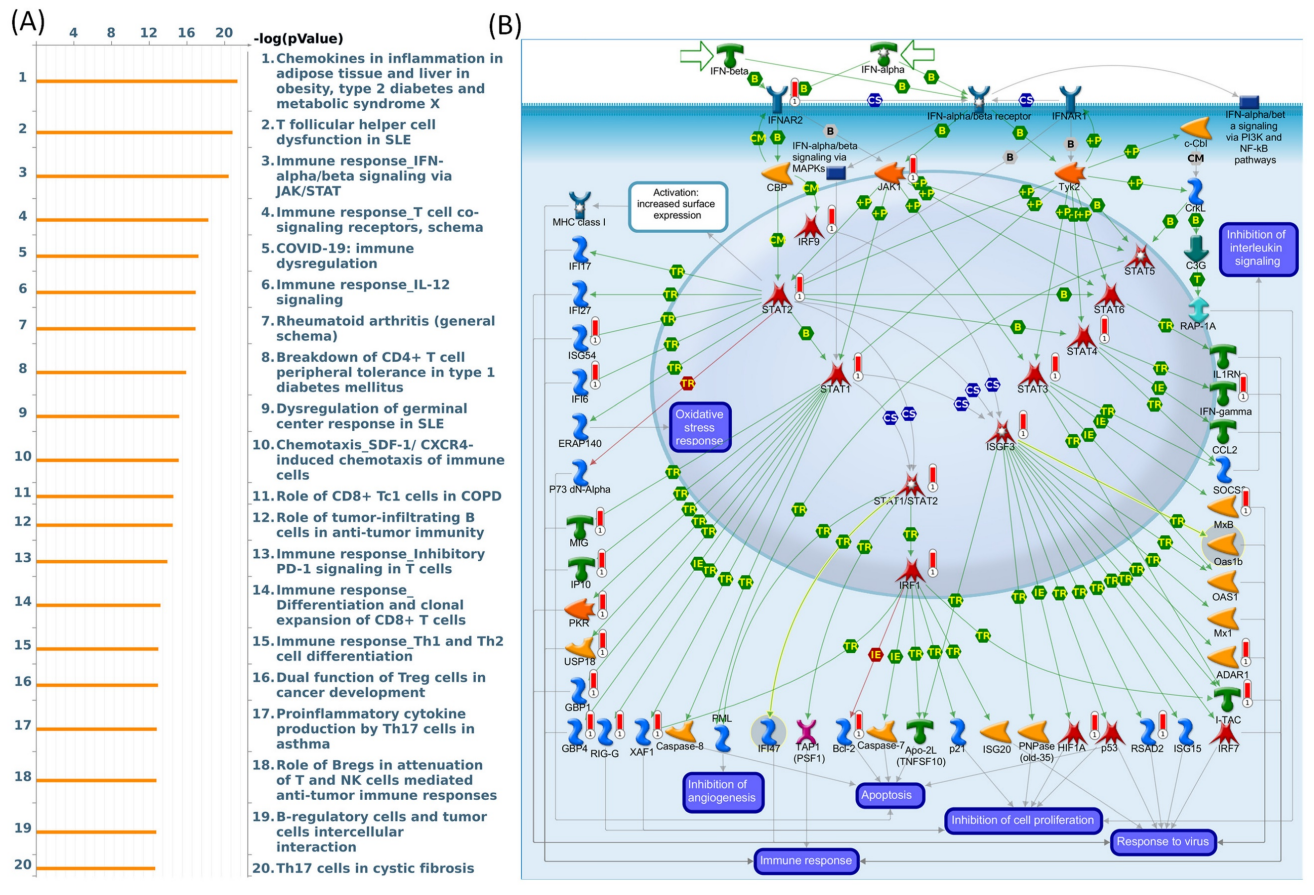
MetaCore pathway analysis of Schlafen 5 (*SLFN5*) co-expressed genes in COAD patients from TCGA. (A) The MetaCore pathway enrichment analysis was conducted for genes co-expressed with *SLFN5* in COAD patients, revealing potential pathways involving these genes ranked by their log *p* values. (B) The "Immune response_IFN-alpha/beta signaling via JAK/STAT" is highlighted, with symbols representing proteins and arrows indicating protein interactions (green for activation and red for inhibition). Thermometer-like histograms visually represent microarray gene expressions, with blue indicating downregulation and red indicating upregulation.

To investigate connections between DEG lists and downstream SLFN5-regulated networks across various biological pathways and diseases, an enrichment analysis was conducted using MetaCore platform. Upon inputting co-expressed genes from the TCGA database into MetaCore, numerous pathways and networks were identified as being associated with metabolism, specifically "Chemokines in inflammation in adipose tissues and liver in obesity, type 2 diabetes and metabolic syndrome X", "Immune response_IFN-alpha/beta signaling via JAK/STAT networks". This summary encapsulates molecular intricacies involved in the regulation and function of the ISGF3 complex within the broader context of cellular signaling pathways, offering insights into the mechanisms by which cells orchestrate a multifaceted response to environmental challenges (Figure 9). Additional information regarding remaining pathways and network-related data are given in Supplementary data (Supplementary Figure S1-3, Table S1).

### 3.6. Association between SFLN5 and drug sensitivity

**Figure 10:**
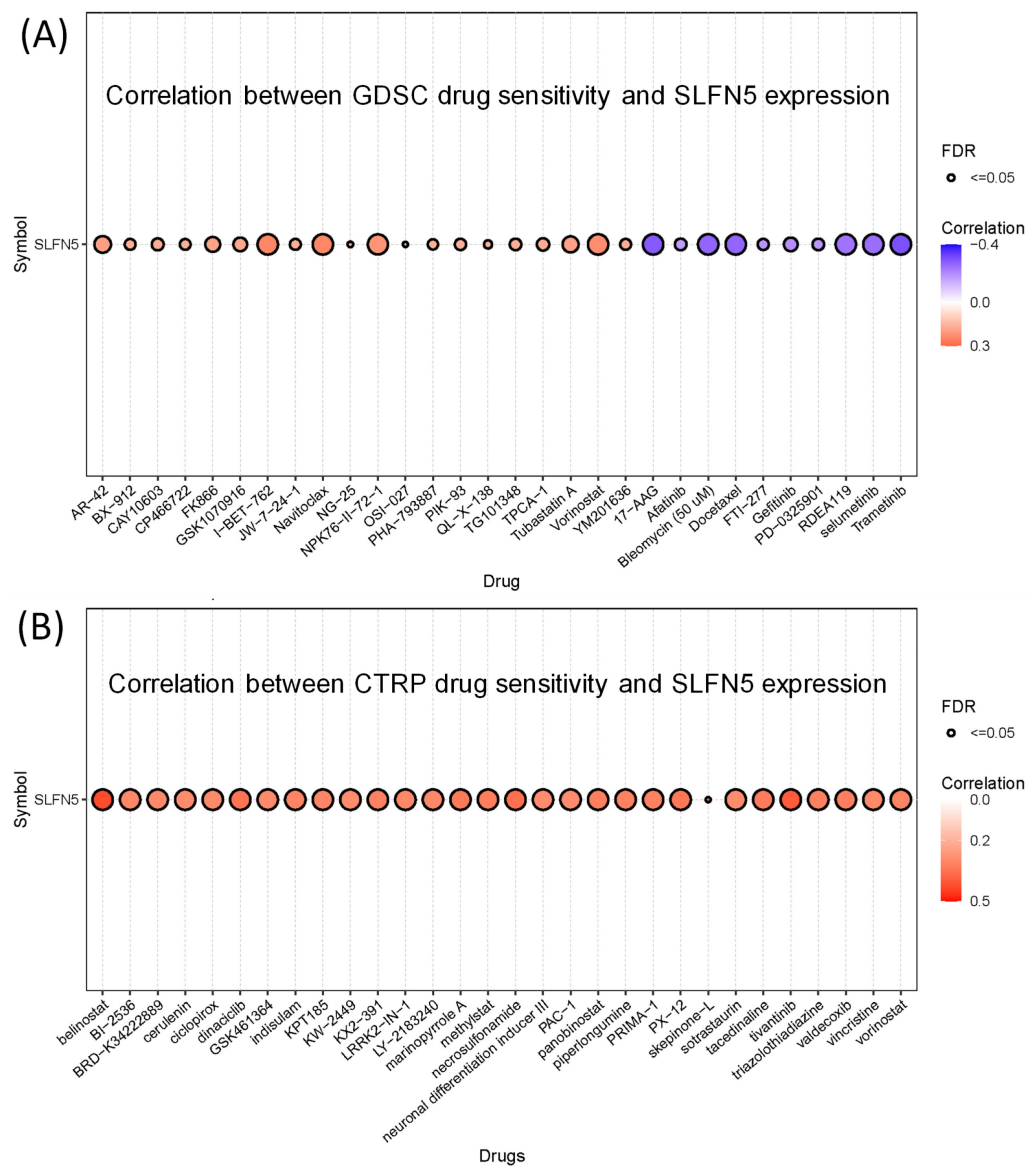
Drug sensitivity of Schlafen 5 (*SLFN5*) oncogene from GSCA. (A) Correlation between Genomics of Drug Sensitivity in Cancer (GDSC) data for FDA-approved drugs according to the *SLFN5* expression levels. (B) Correlation of Cancer Therapeutics Response Portal (CTRP) drug data and *SLFN5*.

In our study, we assessed the correlation between SLFN5 expression and the sensitivity of various drugs using the GSCA database. The results revealed significant correlations for multiple drugs, both positive and negative, indicating how SLFN5 expression levels could potentially influence the efficacy of these treatments (Figure 10).

## 4. Discussion

Despite significant progress in developing new therapies and improving prognoses for COAD patients, a deeper understanding of the molecular signaling pathway underlying these therapeutic effects remains crucial for further treatment optimization. Especially, some patients are diagnosed with advanced stage COAD, characterized by the presence of malignant growth that has already spread to regional lymph nodes. Therefore, there is a critical need to develop innovative biomarker-based prognostic techniques to facilitate early-stage COAD identification 74. Oncogenes are critical genes that contribute to transforming normal cells into malignant ones, and individual pathways characterize tumor genesis and progression. Understanding these aspects may help future anticancer research and prevent cancer development 75. As massive datasets have become available globally, more advanced high-throughput analysis approaches are necessary to analyze these big data. This study sought to identify novel biomarkers, explore molecular pathways, and evaluate treatment modalities as examples of avenues to contribute to the existing knowledge base and potentially improve patient outcomes.

Through SLFN5-regulated networks, the analysis revealed that SLFN5-regulated chemokines are involved in inflammation in adipose tissues and the liver in obesity, type 2 diabetes, and metabolic syndrome X as well as the immune system. These data are consistent with previous research 76, such as platelet endothelial cell adhesion molecule (PECAM)-1 defects being key events for enhanced Akt/glycogen synthase kinase (GSK)-3β signaling in the diabetes mellitus (DM)-associated TME 77. Meanwhile, our pathway enrichment analysis revealed that genes co-expressed with SLFN5 were correlated with "Immune response IFN-alpha/beta signaling via JAK/STAT" in COAD patients. Type I IFNs, via binding to the IFN-alpha/beta receptor, activate the JAK/STAT pathway. STATs and their complexes (INF-stimulated gene factor-3 (ISGF3) or STAT1/STAT2 heterodimers) induce gene expressions required for antiviral (Mx1, adenosine deaminase, RNA-specific (ADAR1), and 2'-5'-oligoadenylate synthetase-1 (OAS1)), anti-proliferative (such as hypoxia-inducible factor-1A (HIF1A)), and immune response (INF-inducible T-cell-alpha chemoattractant (I-TAC), IFN-gamma, and chemokine CC-motif ligand 2 (CCL2)) functions of type I IFNs 78-82.

It is worth mentioning that Tregs play a crucial role in modulating immune responses by suppressing excessive activation of effector T-cell subsets, including T-helper cell type 1 (Th1) Th2, and Th17 cells, thus contributing to the maintenance of intestinal homeostasis 83-85. However, the precise roles and predictive potentials of Tregs in COAD remain ambiguous. In particular, our immune cell deconvolution analysis uncovered correlations between SLFN5 expression and immune-related cell populations, including Tregs, in COAD. Nevertheless, the specific role of SLFN5 in COAD-infiltrating Tregs remains to be elucidated. Further investigation into this relationship may provide valuable insights into the immunoregulatory mechanisms underlying COAD pathogenesis. In detail, our immune cell deconvolution analysis revealed a correlation between SLFN5 expression and immune-related cell populations, such as Tregs. This cascade of events underscores the complexity and specificity of the regulatory mechanisms governing gene transcription in response to external and internal cues, highlighting the intricate interplay between various signaling molecules and transcription factors in cellular defense mechanisms 86-88. Meanwhile, through drug sensitivity results of SFLN5 with potential targeted drugs, which may have therapeutic potential for COAD, and these data are also consistent with previous research.

The present study had several limitations, the study would benefit from additional validation and experimental results to further support and strengthen the findings. We validated our data via IHC assays of COAD clinical patients, however, the limited sample size in this study may restrict the generalizability and statistical power of the observed associations. Despite these limitations, this study established crucial groundwork, identifying avenues for future research and emphasizing the necessity for comprehensive and multifaceted investigations into the involvement of SLFN5 in COAD development.

## 5. Conclusions

This study investigated the role of SLFN5 in COAD. Bulk RNA-sequencing revealed significant overexpression of SLFN5 in COAD tissues compared to normal tissue. Higher SLFN5 expression correlated with poorer overall survival in COAD patients. Immunohistochemistry confirmed elevated SLFN5 protein expression of COAD tissues s compared to normal tissues. Single-cell analysis identified distinct SLFN5 expression patterns across different cell types within the COAD tumor microenvironment. Furthermore, SLFN5 expression correlated with Tregs, suggesting a potential role in immune modulation. DNA methylation analysis revealed specific CpG sites associated with SLFN5 expression. Functional enrichment analyses identified pathways associated with SLFN5 co-expression, including immune response and metabolic regulation. In silico drug sensitivity analysis revealed potential therapeutic drugs targeting SLFN5. These findings highlight SLFN5 as a promising biomarker and therapeutic target for further investigation in COAD.

## Supplementary Material

Supplementary figures and table.
