# Supplementary material for: Comprehensive analysis of bulk and single-cell RNA sequencing data reveals Schlafen-5 (SLFN5) as a novel prognosis and immunity
Source: Int J Med Sci. 2024 Sep 9;21(12):2348–64. doi: 10.7150/ijms.97975 (PMC11413889; doi:10.7150/ijms.97975)
Supplement: Supplementary file 1 — Supplementary figures and table. [file ijmsv21p2348s1.pdf]

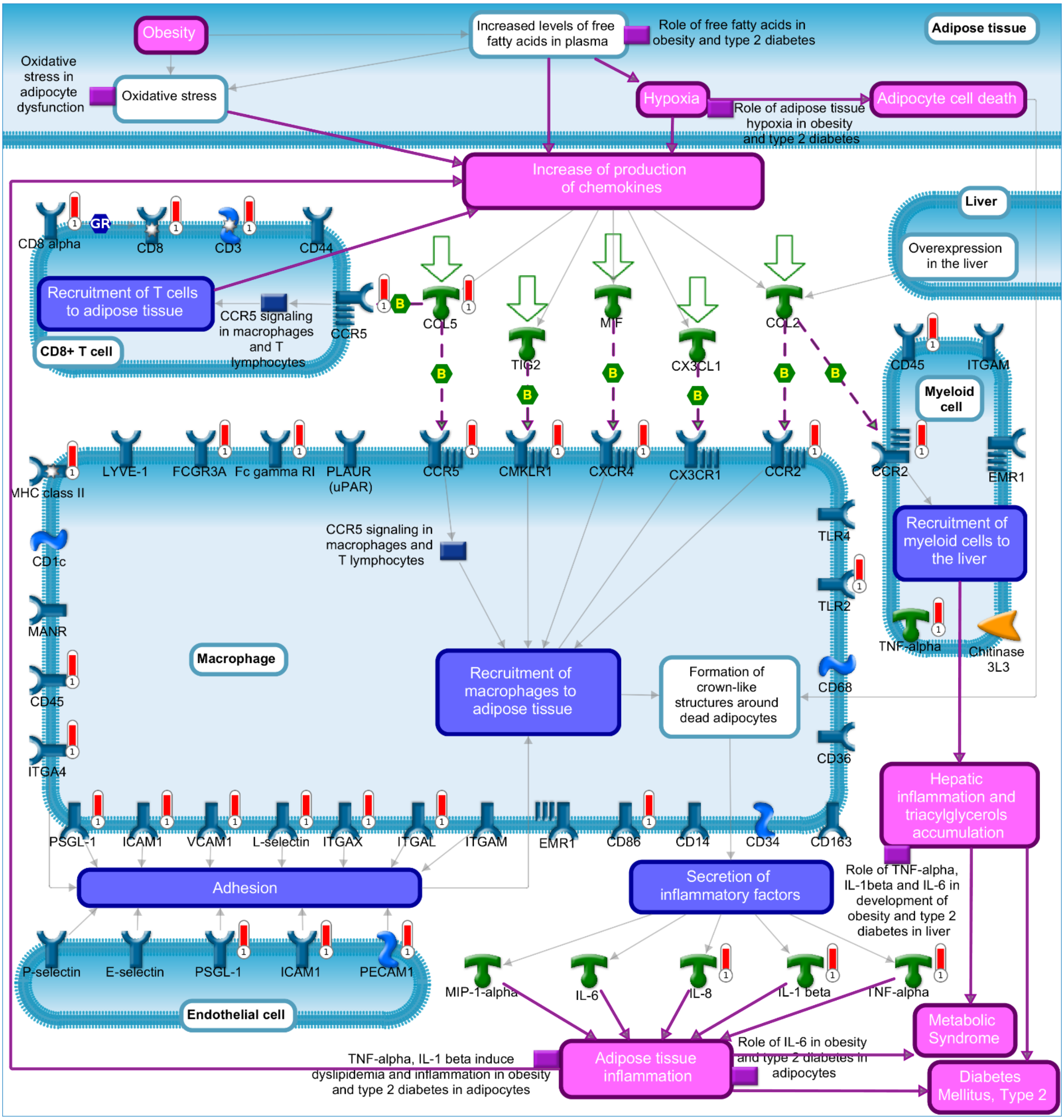

Supplementary Figure S1. MetaCore pathway analysis of Schlafen 5 (SLFN5) co-expressed genes in COAD patients from TCGA. The " Chemokines in inflammation in adipose tissue and liver in obesity, type 2 diabetes and metabolic syndrome X" is highlighted, with symbols representing proteins and arrows indicating protein interactions (green for activation and red for inhibition). Thermometer-like histograms visually represent microarray gene expressions, with blue indicating downregulation and red indicating upregulation.

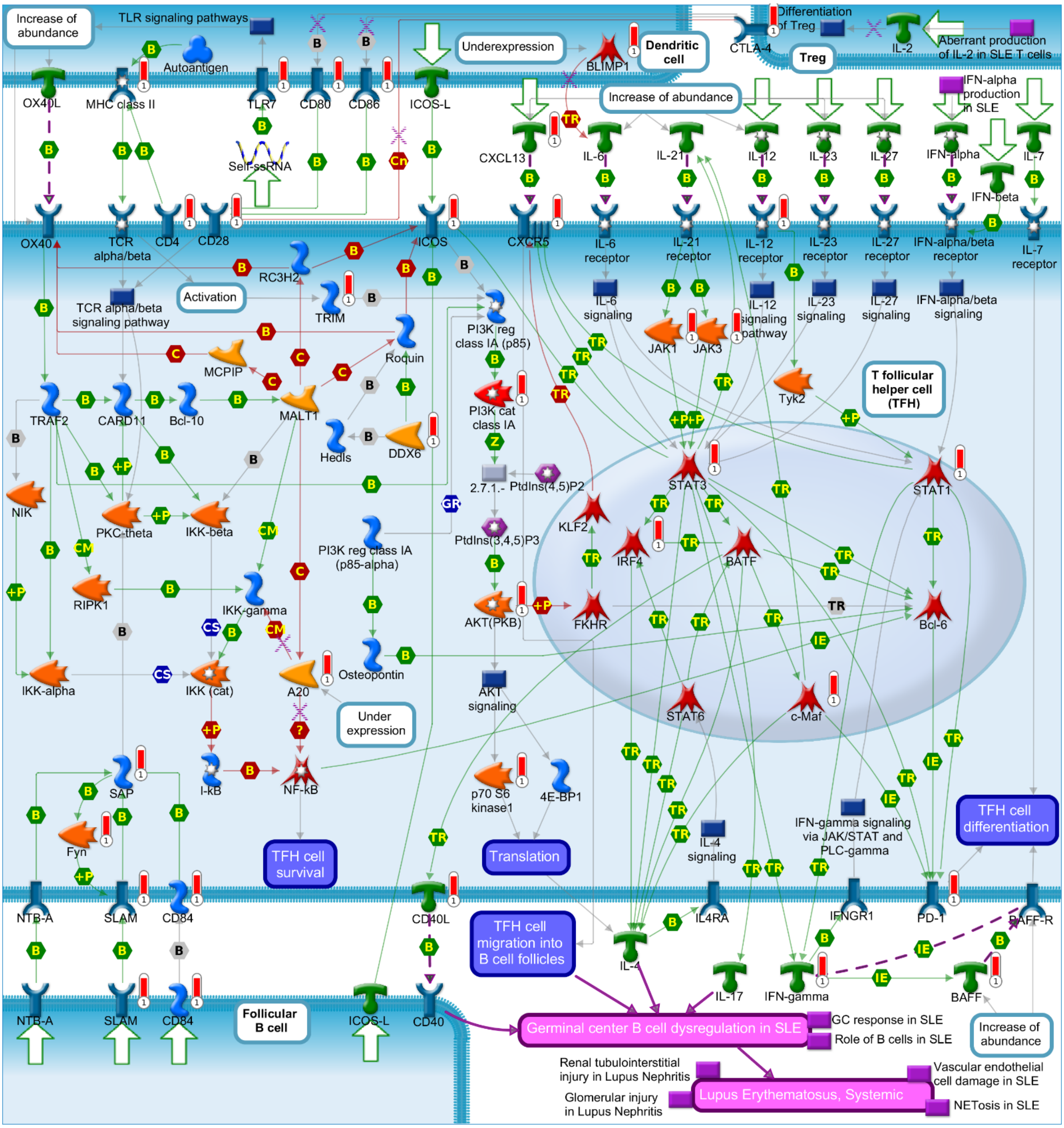

Supplementary Figure S2. MetaCore pathway analysis of Schlafen 5 (SLFN5) co-expressed genes in COAD patients from TCGA. The " T follicular helper cell dysfunction in SLE " is highlighted, with symbols representing proteins and arrows indicating protein interactions (green for activation and red for inhibition). Thermometer-like histograms visually represent microarray gene expressions, with blue indicating downregulation and red indicating upregulation.

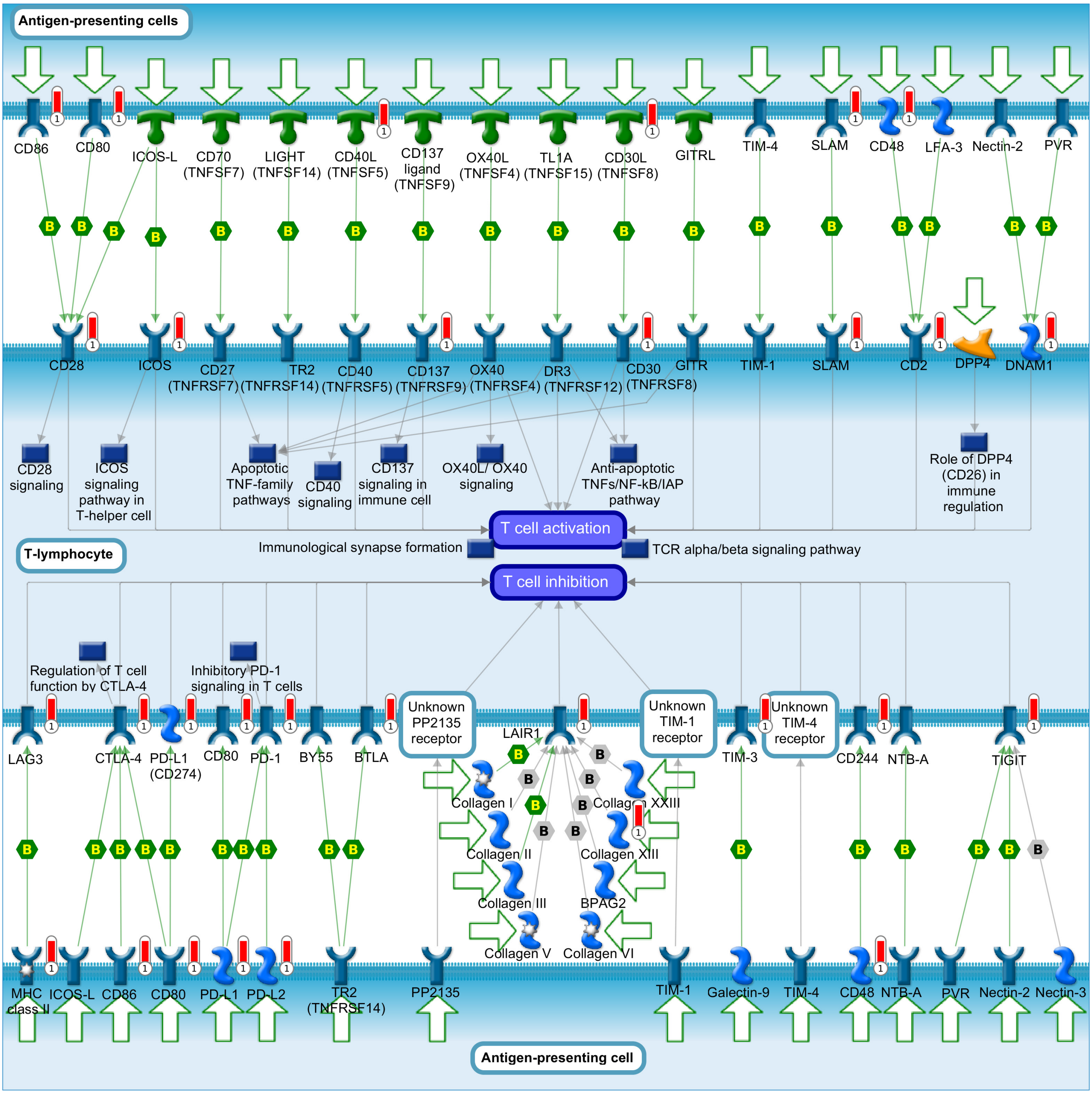

Supplementary Figure S3. MetaCore pathway analysis of Schlafen 5 (SLFN5) co-expressed genes in COAD patients from TCGA. The " Immune response_T cell co-signaling receptors, schema " is highlighted, with symbols representing proteins and arrows indicating protein interactions (green for activation and red for inhibition). Thermometer-like histograms visually represent microarray gene expressions, with blue indicating downregulation and red indicating upregulation.

Supplementary Table S1: Pathway analysis of genes co-expressed SLFN5 from public colorectal cancer databases using the MetaCore database (with p-value < 0.05 set as the cutoff value).

#	Maps	pValue	Network Objects from Active Data
**1**	Chemokines in inflammation in adipose tissue and liver in obesity, type 2 diabetes and metabolic syndrome X	4.24E-22	CD8 alpha, ITGAX, CCL5, IL-1 beta, Fc gamma RI, FCGR3A, L-selectin, PECAM1, MHC class II, VCAM1, CCR5, CD45, ITGA4, CD8, ICAM1, CMKLR1, CD3, ITGAL, IL-8, PSGL-1, CD86, CXCR4, CCR2, TLR2, TNF-alpha
**2**	T follicular helper cell dysfunction in SLE	1.5E-21	CD84, STAT3, CXCR5, CD28, SAP, TRIM, PD-1, CTLA-4, Fyn, CD80, MHC class II, PI3K cat class IA, c-Maf, IL-12 receptor, p70 S6 kinase1, DDX6, SLAM, TLR7, AKT(PKB), JAK1, ICOS, IRF4, A20, JAK3, CD86, IFN-gamma, STAT1, CD4, BLIMP1 (PRDI-BF1), CD40L(TNFSF5), BAFF(TNFSF13B), CXCL13
**3**	Immune response_IFN-alpha/beta signaling via JAK/STAT	3.95E-21	STAT3, MIG, STAT2, GBP1, IRF9, MxB, RSAD2, IP10, GBP4, IRF1, JAK1, STAT4, PKR, HIF1A, IFI6, I-TAC, ISGF3, ISG54, ADAR1, XAF1, IFN-gamma, Bcl-2, STAT1, RIG-G, USP18, IFNAR2, STAT1/STAT2
**4**	Immune response_T cell co-signaling receptors, schema	6.03E-19	DNAM1, Collagen XIII, CD28, CD30L (TNFSF8), TIM-3, PD-1, CTLA-4, CD80, MHC class II, TIGIT, PD-L2, SLAM, CD137(TNFRSF9), BTLA, ICOS, CD48, LAIR1, CD244, CD30(TNFRSF8), CD2, LAG3, CD86, PD-L1, CD40L(TNFSF5)
**5**	COVID-19: immune dysregulation	6.88E-18	CCL5, IL-1 beta, MIP-1-beta, MIG, TIM-3, PD-1, MHC class II, CCR5, TLR7, TLR8, sIL2RA, IP10, CSF1, IRF1, JAK1, CD8, MDA-5, RIG-I, Caspase-1, CD3, HIF1A, ITGAL, IL-8, Granzyme A, Btk, CCR2, IFN-gamma, CD4, HLA-DMA, TNF-alpha
**6**	Immune response_IL-12 signaling	1.36E-17	STAT3, CXCR5, IKBZ, JAK2, PD-1, c-IAP2, Fyn, PI3K cat class IA, IL-12 receptor, AKT(PKB), STAT4, Eomesodermin, ICOS, CD3, IL-8, T-bet, IL-2R alpha chain, CD3 zeta, IL-12RB1, IFN-gamma, Bcl-2, STAT1, BLIMP1 (PRDI-BF1), IL-12RB2, CD40L(TNFSF5), TNF-alpha
**7**	Rheumatoid arthritis (general schema)	1.44E-17	IL-15, IL-1 beta, CD28, Fc gamma RI, TGF-beta, FCGR3A, CD80, MHC class II, VCAM1, PTPN22, CSF1, ICAM1, MHC class II beta chain, IL-2R alpha chain, CD2, CD86, IFN-gamma, TLR2, CD4, CD40L(TNFSF5), BAFF(TNFSF13B), TNF-alpha
**8**	Breakdown of CD4+ T cell peripheral tolerance in type 1 diabetes mellitus	1.54E-16	CXCR5, CD28, PD-1, CTLA-4, CD80, MHC class II, IL-12 receptor, PTPN22, c-FLIP, JAK1, STAT4, ICOS, CD3, T-bet, IL-2R alpha chain, JAK3, CD86, IFN-gamma, CD4, PD-L1, CD40L(TNFSF5)
**9**	Dysregulation of germinal center response in SLE	8.63E-16	STAT3, IL-15, CXCR5, Fc gamma RII beta, IRF9, VCAM1, TLR7, FasL(TNFSF6), Oct-2, CD21, ESR1 (nuclear), JAK1, ICAM1, IRF4, HOXC4, JAK3, BOB1, PI3K cat class IA (p110-

			delta), STAT1, CD40L(TNFSF5), BAFF(TNFSF13B), CXCL13, DOCK8
**10**	Chemotaxis_SDF-1/ CXCR4-induced chemotaxis of immune cells	9.86E-16	RASSF5, G-protein beta/gamma, JAK2, WASP, Fyn, PKA-reg (cAMP-dependent), PI3K cat class IA, VCAM1, p70 S6 kinase1, CD45, AKT(PKB), ICAM1, CD3, SFK, PI3K reg class IB (p101), PLC-gamma 2, CD3 zeta, ITK, JAK3, Btk, VAV-1, CXCR4, ROCK1, PI3K cat class IB (p110-gamma), Cofilin
**11**	Role of CD8+ Tc1 cells in COPD	3.68E-15	CD69, STAT3, MMP-12, IL-15, JAK2, MIG, IL-18R1, IL-12 receptor, CCR5, IP10, CD8, STAT4, KLRK1 (NKG2D), Cathepsin S, T-bet, CXCR6, IFN-gamma, Bcl-2, TNF-alpha
**12**	Role of tumor-infiltrating B cells in anti-tumor immunity	4.22E-15	STAT3, CXCR5, G-protein beta/gamma, MIG, IL-18R1, MHC class II, IL-12 receptor, FasL(TNFSF6), IP10, CD20, JAK1, CD8, IRF4, CD38, I-TAC, RGS1, T-bet, JAK3, Btk, CXCR4, IFN-gamma, STAT1, CD4, BLIMP1 (PRDI-BF1), CD40L(TNFSF5), CXCL13
**13**	Immune response_Inhibitory PD-1 signaling in T cells	1.58E-14	CD8 alpha, CD28, PD-1, CD80, MHC class II, PI3K cat class IA, PD-L2, FYB1, AKT(PKB), CD8, ICAM1, PTEN, Eomesodermin, CD3, T-bet, CD3 zeta, CD86, VAV-1, CD4, PD-L1
**14**	Immune response_Differentiation and clonal expansion of CD8+ T cells	8.78E-14	STAT3, CD28, CD80, MHC class II, IL-12 receptor, FasL(TNFSF6), CD8, ICAM1, CD137(TNFRSF9), STAT4, T-bet, CD86, IFN-gamma, STAT1, CD4, IL-12RB2, CD40L(TNFSF5)
**15**	Immune response_Th1 and Th2 cell differentiation	1.47E-13	CD28, JAK2, CD80, MHC class II, c-Maf, IL-12 receptor, JAK1, CD137(TNFRSF9), STAT4, CD3, T-bet, CD86, IFN-gamma, STAT1, CD4, IL-12RB2, CD40L(TNFSF5)
**16**	Dual function of Treg cells in cancer development	1.58E-13	STAT3, Helios, TIM-3, PD-1, STAT2, CTLA-4, c-Maf, FasL(TNFSF6), CD45, ROR-alpha, ICOS, ENP1, ETS1, IL-2R alpha chain, IFN-gamma, Neuropilin-1, STAT1, CD4
**17**	Proinflammatory cytokine production by Th17 cells in asthma	2.17E-13	STAT3, IL-1RI, IL-1 beta, CD28, JAK2, CD80, MHC class II, ROR-alpha, JAK1, IRF4, IL23A, IL-8, IL-22, JAK3, CCR4, CD86, CXCR4, CD4, TNF-alpha
**18**	Role of Bregs in attenuation of T and NK cells mediated anti-tumor immune responses	2.41E-13	STAT3, CD28, MIG, PD-1, CTLA-4, L-selectin, CD80, FasL(TNFSF6), IP10, CD3, ENP1, IL-2R alpha chain, CD3 zeta, LAG3, CD86, IFN-gamma, PD-L1
**19**	B-regulatory cells and tumor cells intercellular interaction	2.49E-13	STAT3, IL-1RI, IL-1 beta, CD28, LTB, JAK2, PD-1, CD80, c-Maf, VEGFR-2, TLR7, FasL(TNFSF6), JAK1, JAK3, CD86, CCR2, STAT1, PD-L1, IL-12RB2, CD40L(TNFSF5), TNF-alpha
**20**	Th17 cells in cystic fibrosis	3.22E-13	STAT3, IL-1RI, IL-1 beta, CD28, JAK2, CD80, MHC class II, VCAM1, GCP2, ROR-alpha, JAK1, ICAM1, IRF4, IL23A, IL-8, JAK3, CD86, CD4, MD-2

**21**	Chemotaxis_Common mechanisms of Th17 cell migration	3.78E-13	CCL5, CD161, MIP-1-beta, G-protein beta/gamma, MIG, VCAM1, CCR5, IP10, CD43, ICAM1, I-TAC, PI3K reg class IB (p101), PSGL-1, CXCR6, CCR4, CCR2, PI3K cat class IB (p110-gamma), IP3 receptor
**22**	Immune response_PIP3 signaling in B lymphocytes	3.89E-13	Fc gamma RII beta, PKC-beta, G-protein beta/gamma, PI3K cat class IA, p70 S6 kinase1, CD45, BCAP, AKT(PKB), DAPP1, PTEN, PI3K reg class IB (p101), Lyn, Btk, VAV-1, PLC-gamma, PI3K cat class IB (p110-gamma), IP3 receptor
**23**	Glomerular injury in Lupus Nephritis	4.44E-13	CCL5, IL-1 beta, TGF-beta, VCAM1, FasL(TNFSF6), PKC-beta1, IP10, AKT(PKB), CSF1, IRF1, ICAM1, MDA-5, RIG-I, HIF1A, CaMK IV, IL-8, NGAL, A20, IFI56, PKC-beta2, IFN-gamma, Bcl-2, STAT1, TNF-alpha
**24**	Immune response_Immunological synapse formation	4.72E-13	RASSF5, CD28, WASP, Fyn, CD80, MHC class II, PI3K cat class IA, VCAM1, Slp76, FYB1, PREL1, ICAM1, CD3, GRAP2, Cytohesin1, ITK, CD86, VAV-1, WaspIP
**25**	Immune response_IL-10 signaling	5.03E-13	STAT3, IL-1 beta, Heme oxygenase 1, Fc gamma RI, Fc gamma RII beta, IKBZ, CD80, MHC class II, PI3K cat class IA, p70 S6 kinase1, AKT(PKB), Fc gamma RII alpha, JAK1, IL10RA, ICAM1, IL-8, CD86, Bcl-2, STAT1, TNF-alpha
**26**	CHDI_Correlations from Replication data_Causal network (positive correlations)	9.96E-13	IL-1RI, CD28, IP3R1, G-protein beta/gamma, ROCK, CD80, MHC class II, PI3K cat class IA, CD83, MEF2, CD45, Slp76, AKT(PKB), ICAM1, CD3, CaMK IV, PI3K reg class IB (p101), ITK, CXCR4, TLR2, PI3K cat class IB (p110-gamma), IP3 receptor
**27**	NK cells in allergic contact dermatitis	2.09E-12	CIITA, CCL5, MIG, MHC class II, CCR5, FasL(TNFSF6), IP10, IRF1, ICAM1, KLRK1 (NKG2D), NKp46, CCR2, IFN-gamma, STAT1, TNF-alpha
**28**	Immune response_Naive CD4+ T cell differentiation	2.28E-12	STAT3, IL-1 beta, CD28, CD80, MHC class II, c-Maf, ROR-alpha, STAT4, IRF4, IL-22, T-bet, CD86, IFN-gamma, STAT1, CD4, CD40L(TNFSF5), TNF-alpha
**29**	Immune response_ICOS signaling in T-helper cell	2.82E-12	CXCR5, CD28, Tiam1, ROCK, MHC class II, PI3K cat class IA, c-Maf, p70 S6 kinase1, AKT(PKB), ICOS, CD3, T-bet, ITK, VAV-1, IFN-gamma, IP3 receptor, CD4, CD40L(TNFSF5), TNF-alpha
**30**	Immune response_IL-16 signaling	3.95E-12	CCL5, IL-15, IL-2R beta chain, IL-16 mature, IL-1 beta, MLCP (reg), MIP-1-beta, cPKC (conventional), CCR5, IL-16, PKC, AKT(PKB), N-terminal IL16, IL-2R alpha chain, CXCR4, IP3 receptor, CD4, TNF-alpha
**31**	Immune response_Glucocorticoid receptor immunological signaling	6.42E-12	CIITA, CCL5, IL-1 beta, MIP-1-beta, MIG, PD-1, PI3K cat class IA, IL7RA, TIGIT, IP10, AKT(PKB), ICAM1, GCR, IL-8, T-bet, IFI56, CXCR4, IFN-gamma, BLIMP1 (PRDI-BF1), TNF-alpha

**32**	Immune response_CD40 signaling in dendritic cells, monocytes, and macrophages	6.75E-12	IL-1RI, IL-1 beta, IDO1, TPL2(MAP3K8), c-IAP2, p70 S6 kinases, CD80, MHC class II, PI3K cat class IA, CD83, PKC-beta1, AKT(PKB), IL-8, Lyn, CalDAG-GEFII, JAK3, PKC-beta2, CD86, VAV-1, CD40L(TNFSF5), TNF-alpha
**33**	CD8+ Tc1 cells in allergic contact dermatitis	8.56E-12	CD8 alpha, IL-1RI, IL-1 beta, CD28, CD80, MHC class II, FasL(TNFSF6), CD8, Caspase-1, CD86, IFN-gamma, CD4, TNF-alpha
**34**	Immune response_Antimicrobial actions of IFN-gamma	9.02E-12	IFP 35, MIP-1-beta, JAK2, IDO1, SLC26A3 (DRA), GBP1, p67-phox, IRF1, JAK1, RIG-I, NGAL, IFN-gamma, STAT1, Staf-50, GBP2, MD-2
**35**	Modulation of tumor response to cytotoxic T cells by hypoxia in tumors	1.09E-11	STAT3, CD28, NIX, JAK2, PD-1, CTLA-4, CD80, VEGFR-2, FasL(TNFSF6), AKT(PKB), CD8, CD137(TNFRSF9), HIF1A, ENP1, CD86, Bcl-2, PD-L1
**36**	Immune response_TCR alpha/beta signaling	1.19E-11	CD28, P2X7, WASP, Fyn, c-FLIP(Long), CD80, MHC class II, CD45, Slp76, FYB1, AKT(PKB), CD8, CD3, GRAP2, CaMK IV, CalDAG-GEFII, ITK, CD86, VAV-1, IP3 receptor, CD4, CFLAR p43, WaspIP
**37**	Differences between Langerhans cells and dermal dendritic cells in allergic contact dermatitis	1.44E-11	CD28, PD-1, CD80, MHC class II, CD8, ICOS, CD86, CXCR4, CD4, PD-L1, CD40L(TNFSF5), TNF-alpha
**38**	SLE genetic marker-specific pathways in B cells	1.86E-11	BLK, IP3R1, Fc gamma RII beta, STAT2, BANK, IRF9, Ikaros, TLR7, p67-phox, CalDAG-GEFIII, CD38, ETS1, Lyn, Aiolos, Btk, VAV-1, PLC-gamma, Bcl-2, STAT1, IP3 receptor, BLIMP1 (PRDI-BF1), BAFF(TNFSF13B), TNF-alpha
**39**	Immune response_Antiviral actions of Interferons	2.25E-11	CIITA, JAK2, IDO1, MxA, STAT2, OAS3, MHC class II, IRF9, OAS2, IRF1, JAK1, PKR, ISGF3, ADAR1, IFN-gamma, 2'-5'-oligoadenylate synthetase, STAT1
**40**	Immune response_T cell subsets: cell surface markers	2.25E-11	IL-1RI, CD161, CTLA-4, L-selectin, CD94, IL-12 receptor, IL7RA, CCR5, SLAM, CD8, BTLA, CD3, IL-2R alpha chain, CCR4, LAG3, CD4, IL-12RB2
**41**	SLE genetic marker-specific pathways in T cells	2.89E-11	STAT3, IP3R1, PD-1, MHC class II, IL-12 receptor, Ikaros, PTPN22, Slp76, CalDAG-GEFIII, ESR1 (nuclear), MHC class II beta chain, STAT4, CD3, KLRK1 (NKG2D), ETS1, T-bet, Aiolos, CD3 zeta, A20, VAV-1, IFN-gamma, Bcl-2, CD40L(TNFSF5)
**42**	Role of IL-23/ T17 pathogenic axis in psoriasis	3.18E-11	STAT3, IL-15, IL-1RI, IL-2R beta chain, IL-1 beta, CD28, CD80, MHC class II, AKT(PKB), Calgranulin A, IRF4, IL23A, IL-8, IL-22, CD86, CD4, CD40L(TNFSF5)
**43**	Memory CD8+ T cells in allergic contact dermatitis	3.57E-11	CCL5, IL-15, IL-1RI, IL-2R beta chain, IL-1 beta, IL7RA, VCAM1, CD8, ICAM1, Eomesodermin, PSGL-1, T-bet, CCR4, IFN-gamma, TNF-alpha

**44**	Immune response_Th17 cell differentiation	5.8E-11	STAT3, IL-1RI, IL-1 beta, CD28, JAK2, CD80, MHC class II, ROR-alpha, IRF4, IL23A, IL-22, JAK3, CD86, CD4
**45**	Th17 cells in cystic fibrosis (mouse model)	8.97E-11	STAT3, IL-1RI, IL-1 beta, CD28, JAK2, CD80, MHC class II, GCP2, JAK1, ICAM1, IRF4, IL23A, JAK3, CD86, CD4, MD-2
**46**	Th17 cytokines in COPD	9.12E-11	STAT3, MMP-12, IL-1 beta, CD28, CD80, MHC class II, IP10, ROR-alpha, CD8, IL-8, IL-22, CD86, CCR2, CD4
**47**	Immune response_IL-22 signaling	9.12E-11	STAT3, CD28, JAK2, MHC class II, IL-12 receptor, JAK1, STAT4, Mcl-1, IL-22, JAK3, CD86, Bcl-2, STAT1, CD4
**48**	Immune response_Antigen presentation by MHC class I: cross-presentation	1.3E-10	Cathepsin L, CLEC12A, Dectin-1, Fc gamma RI, Rab-27A, TIM-3, SREC-I, FCGR3A, TLR7, CD74, p67-phox, Fc gamma RII alpha, LLIR, CD8, Rab8B, Fc epsilon RI gamma, Cathepsin S, VAV-1, TLR2, gp91-phox, CD209, p47-phox
**49**	Immune response_Generation of memory CD4+ T cells	1.41E-10	IL-15, CD28, BFL1, L-selectin, CD80, MHC class II, IL7RA, JAK1, Mcl-1, IL-2R alpha chain, JAK3, CD86, Bcl-2, CD4
**50**	Role of B cells in SLE	1.59E-10	CD28, Fc gamma RII beta, CD80, MHC class II, PI3K cat class IA, CD45, AKT(PKB), ICAM1, ICOS, Lyn, Btk, CD86, IFN-gamma, Bcl-2, CD40L(TNFSF5), BAFF(TNFSF13B), TNF-alpha
